# Medication audit and feedback by a clinical pharmacist decrease medication errors at the PICU: An interrupted time series analysis

**DOI:** 10.1002/hsr2.23

**Published:** 2018-01-19

**Authors:** Jolanda M. Maaskant, Marieke A. Tio, Reinier M. van Hest, Hester Vermeulen, Vincent G.M. Geukers

**Affiliations:** ^1^ Department of Pediatric Intensive Care, Emma Children's Hospital Academic Medical Center Amsterdam The Netherlands; ^2^ Department of Clinical Epidemiology, Biostatistics, and Bioinformatics, Medical Faculty Academic Medical Center and University of Amsterdam Amsterdam The Netherlands; ^3^ ACHIEVE Centre of Applied Research, Faculty of Health Amsterdam University of Applied Sciences Amsterdam The Netherlands; ^4^ Department of Hospital Pharmacy Academic Medical Center Amsterdam The Netherlands; ^5^ Radboud Institute for Health Sciences, Scientific Center for Quality of Healthcare (IQ Healthcare) Radboud University Medical Center Nijmegen The Netherlands

**Keywords:** harm, ITS, medication error, multifaceted intervention, pharmacist, PICU

## Abstract

**Objective:**

Medication errors (MEs) are one of the most frequently occurring types of adverse events in hospitalized patients and potentially more harmful in children than in adults. To increase medication safety, we studied the effect of structured medication audit and feedback by a clinical pharmacist as part of the multidisciplinary team, on MEs in critically ill children.

**Method:**

We performed an interrupted time series analysis with 6 preintervention and 6 postintervention data collection points, in a tertiary pediatric intensive care unit. We included intensive care patients admitted during July to December 2013 (preintervention) and July to December 2014 (postintervention). The primary endpoint was the prevalence of MEs per 100 prescriptions. We reviewed the clinical records of the patients and the incident reporting system for MEs. If an ME was suspected, a pediatrician‐intensivist and a clinical pharmacist determined causality and preventability. They classified MEs as harmful according to the National Coordinating Council for Medication Error Reporting and Prevention categories.

**Results:**

We included 254 patients in the preintervention period and 230 patients in the postintervention period. We identified 153 MEs in the preintervention period, corresponding with 2.27 per 100 prescriptions, and 90 MEs in the postintervention period, corresponding with 1.71 per 100 prescriptions. Autoregressive integrated moving average analyses revealed a significant change in slopes between the preintervention and postintervention periods (β = −.21; 95% CI, −0.41 to −0.02; P = .04). We did not observe a significant decrease immediately after the start of the intervention (β = −.61; 95% CI, −1.31 to 0.08; P = .07).

**Conclusion:**

The implementation of a structured medication audit, followed by feedback by a clinical pharmacist as part of the multidisciplinary team, resulted in a significant reduction of MEs in a tertiary pediatric intensive care unit.

## Abstract 2

Our study shows that the implementation of structured medication audit, followed by timely feedback by a clinical pharmacist as part of the multidisciplinary team, resulted in a significant reduction of medication errors in a tertiary pediatric intensive care unit. We observed a nonsignificant decrease in medication‐related patient harm. The proactive role of the clinical pharmacist resulted in recommendations with a high acceptance rate.

## 1 INTRODUCTION

Medication errors (MEs) are among the most frequently occurring types of adverse events in hospitalized patients, and 3% to 10% of MEs result in patient harm.^1^, ^2^, ^3^ Medication errors are also associated with additional costs up to $8.500 per patient, as estimated for hospitals in the United States.^4^ The reported prevalence of MEs varies from 5 to 24 per 100 prescriptions in pediatric inpatients.^5^, ^6^, ^7^, ^8^ A previous study suggested that MEs are potentially more harmful in children than in adults.^6^ Children admitted to pediatric intensive care units (PICUs) are especially vulnerable to harmful MEs because of their dependence on multiple and life‐supporting medications.^9^

Because of the growing awareness of the complexity of the medication process and medication safety issues, it has been suggested that active involvement of a clinical pharmacist on pediatric wards might be of additional value. Three systematic reviews report a reduction of MEs after a pharmacist was employed on clinical wards, but the included studies do not provide a clear description of the interventions by the clinical pharmacist.^10^, ^11^, ^12^ In addition, quality issues arise as most of the included publications involved observational studies, before‐and‐after designs were without a control group, or the MEs were self‐reported by the intervening pharmacist.^10^, ^11^, ^12^ A recent Cochrane systematic review^13^ included only 1 high‐quality, controlled before‐after study that showed a significant reduction of serious MEs after the implementation of a multifaceted intervention by a full‐time clinical pharmacist on a PICU.^14^

Since available evidence is scarce, we decided to study the effect of a structured audit of prescribed medication, followed by feedback to the prescribing pediatrician‐intensivist and bedside nurse by a clinical pharmacist as part of the multidisciplinary PICU team. We formulated the following research questions:

- Do MEs and medication‐related patient harm on a PICU decrease after the implementation of a structured medication audit, followed by feedback from a clinical pharmacist as part of a multidisciplinary team?
- What types of recommendations are made by the clinical pharmacist and to what extent are they accepted by the medical and nursing staff?

## 2 METHODS

The Institutional Review Board of the Academic Medical Center in Amsterdam ascertained that medical ethical approval was not required. All patients were informed about the fact that health‐related data collected routinely could be used for quality improvement, evaluation of care, and scientific research. Patients were given the opportunity to refuse. All data were analyzed and reported anonymously. This is in line with the research code at the Amsterdam Medical Center, and it complies with Dutch Medical Ethics Law.

### 2.1 Setting and study population

We performed our study in the tertiary PICU of Emma Children's Hospital/Academic Medical Center, Amsterdam, The Netherlands. This mixed PICU has a capacity of 12 beds and provides care to approximately 600 intensive care patients and 300 high‐care patients annually, ranging in age from newborns to 18 years.

At the time of the study, all medications were prescribed or altered during the morning round, using a stand‐alone patient data management system (PDMS). This PDMS is a generic ordering system and is not equipped with a medication safety monitoring or decision support system. At the start of every nursing shift, an electronically generated sign‐off medication list was printed for every patient separately. Electronic alterations could be made to the medication list by the attending resident, fellow, or staff member. After a mandatory double check, the prescribed medications were administered to the patient, and both nurses signed off the medications on the list. Guidelines of all medications were available on the ward in a hospital formulary.

We included all intensive care patients with at least 1 medication prescription and with an expected length of stay in the PICU of more than 24 hours. We excluded high‐care patients from our study.

### 2.2 Study design and endpoints

We performed an interrupted time series (ITS) with 6 preintervention and 6 postintervention data collection points. We considered 1‐month intervals between data collection points as adequate to identify trends in the occurrence rate of MEs. For accurate comparison of the preintervention and postintervention data, the data collection took place during the same calendar months of 2 consecutive years to rule out seasonal effect. The primary endpoint was the prevalence of MEs per 100 prescriptions. Secondary outcomes were medication‐related patient harm per 100 prescriptions, the types of the recommendations by the clinical pharmacist, and their acceptance by the clinicians.

We used the definitions and categories for error and harm as described by the National Coordinating Council for Medication Error Reporting and Prevention^15^ (Appendix S1). High‐alert medications were recorded according to the list for pediatric patients.^16^ A prescription was defined as a recipe written by the pediatrician‐intensivist to start or change medication, including change of dose.

### 2.3 Interventions by the clinical pharmacist

The study intervention was the expansion of the PICU team with a clinical pharmacist. The clinical pharmacists received mandatory training before the implementation period on the PICU started. During their training, they familiarized themselves with prevailing medication protocols and guidelines and with data collection from the electronic hospital systems, including the PDMS.

The clinical pharmacist was present on the PICU for a maximum of 3 hours every morning from Monday through Friday. At the beginning of the workday, patients considered most at risk for MEs were selected for the medication audit by the attending pediatrician‐intensivist together with the clinical pharmacist using the following criteria: (*a*) reduced renal and/or hepatic clearance, (*b*) oncological diagnoses, (*c*) high‐alert medication prescriptions, (*d*) receiving more than 5 medications, and (*e*) medication prescriptions with which the PICU professionals felt unfamiliar. The clinical pharmacist performed a structured audit of the prescribed medication for the selected patients, followed by feedback and recommendations to the attending pediatrician‐intensivist and nurse during the ward round later the same morning. Administration of medication was discussed with the bedside nurse, eg, compatibility of medication administration, and infusion pump rates. A structured form was used for the medication audit and bedside evaluation (Appendix S2).

### 2.4 Data collection

**Figure 1:**
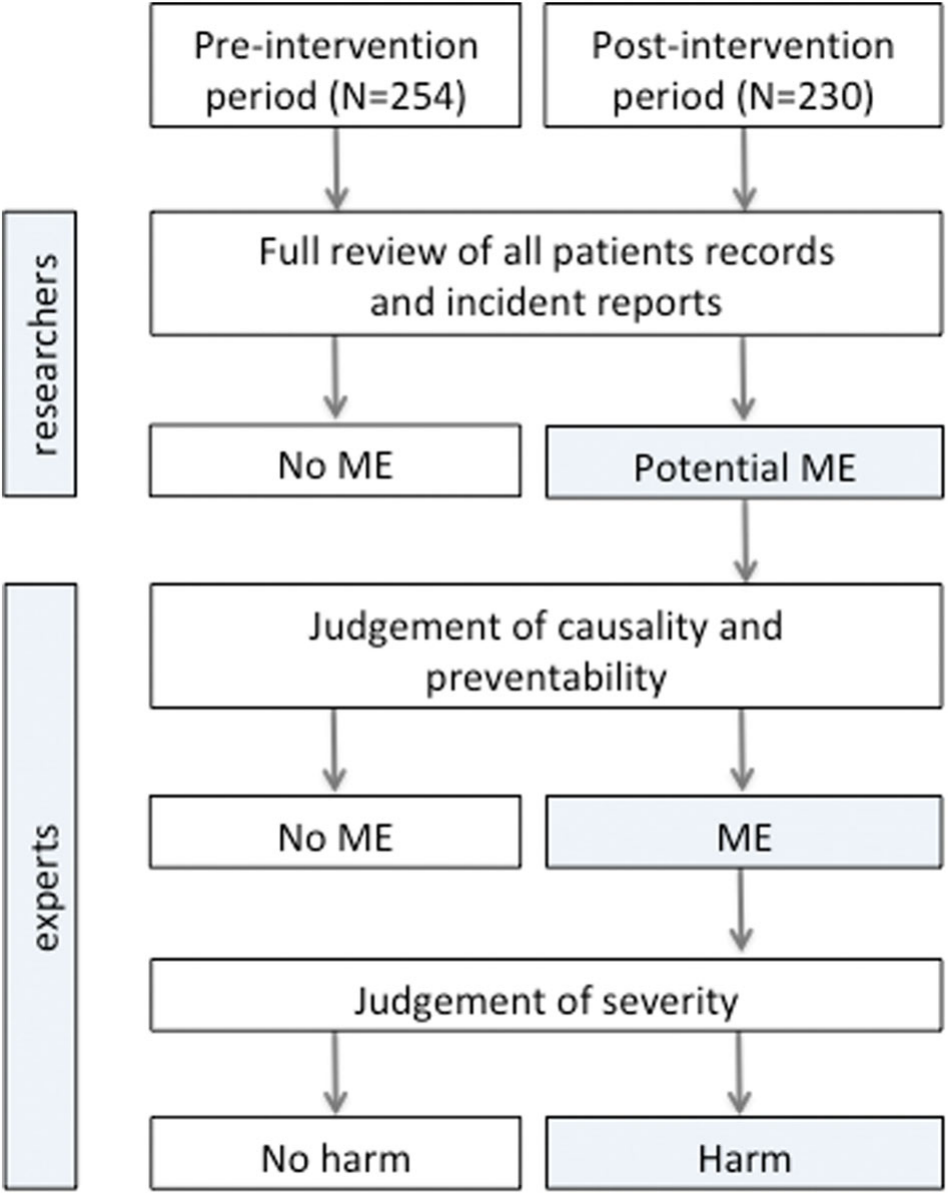
Flowchart data collection. ME, medication errors

Data on MEs and patient harm were collected for all included patients, ie, both the patients who were audited by the clinical pharmacist and the nonaudited patients. To establish the prevalence of MEs and patient harm, we used a 3‐step approach that was validated in a previous study.^17^ During the first step, the clinical records of discharged patients were retrospectively reviewed by one of the investigators (J. M. or M. T.). Potential MEs were identified by reviewing all medication summaries, check‐off lists, medical and nursing daily notes, symptom registration, and postoperative notes. We systematically compared the potential MEs with the local protocols and the Dutch pediatric formulary.^18^ In addition, the hospital incident reporting system was reviewed for reported MEs during the study period. During the second step, we presented the identified potential MEs to a blinded pediatrician‐intensivist and a clinical pharmacist, who deemed the identification of potential MEs to be true or false. In the third step, they classified the MEs as harmful according to the National Coordinating Council for Medication Error Reporting and Prevention categories. The process of data collection is visualized in Figure 1.

Every day during the postintervention period, the clinical pharmacists registered information on the recommendations and the acceptance on the structured medication audit form. Acceptance was scored positively when a recommendation was followed up within 24 hours.

The data collection on MEs and potential patient harm was performed by 2 researchers (J. M. and M. T.). Data were collected on paper on self‐designed forms and were then transferred electronically (J. M.). During the collection of data on all MEs in the postintervention period, the researchers (J. M. and M. T.) and the experts (V. G. and R. v. H.) were blinded for the patients selected for the medication audits.

Two researchers (J. M. and M. T.) collected the data in parallel from the first month of the preintervention period independently, and discrepancies were discussed until consensus was reached. During the other study data collection periods, the investigators performed double checks on the patient files that were considered complex by discretion of the researchers.

### 2.5 Power calculation and statistical analyses

We estimated a prevalence of 10 MEs per 100 prescriptions in the preintervention group and 3 MEs per 100 prescriptions in the postintervention group. With a type 1 error of 0.05 and a power of 0.80, we required a sample size of 237 patients per group. Descriptive statistics were used to summarize patient demographics and the recommendations of the clinical pharmacists. If normally distributed, continuous values were expressed as mean with standard deviation; in case of nonnormal distribution, data were expressed as median with interquartile range. Chi‐squared analysis, the Mann‐Whitney test, or the unpaired Student *t* test was used to compare the preintervention and postintervention characteristics of patients and medications. Error rates were plotted over time to examine the data visually, and we used autoregressive integrated moving average ITS techniques to study the effect of the intervention. Statistical uncertainty was expressed by 95% confidence interval and a *P* value of .05 was considered statistically significant. All analyses were performed using the SPSS software (PASW statistics version 22.0, IBM, Armonk, NY).

## 3 RESULTS

### 3.1 Patients and prescriptions

Patients were included from 1 July 2013 until 31 December 2013 (preintervention) and from 1 July 2014 until 31 December 2014 (postintervention). In total, 254 patients in the preintervention period and 230 patients in the postintervention period met the inclusion criteria of the study and were included in the analyses. Seven patients were excluded owing to missing files. Our total study population represented 1915 admission days, during which 11 995 prescriptions were written and 28 496 doses of medicine were administered. There were significantly more patients with more than 5 prescriptions in the postintervention period compared with the preintervention period (80% and 88%, respectively, *P* = .02). The patients' characteristics are summarized in Table 1.

**Table 1:** Patients' characteristics

Characteristic	Preintervention	Postintervention	*P* Value
	n = 254	n = 230	
Demographics			
Male, n (%)	143 (56)	133 (58)	.74
Age in months, median (IQR)	32.5 (98)	35.0 (106)	.37
Severity of illness			
PRISM III, median (IQR)	2.5 (5)	3.0 (7)	.06
Invasive ventilation, n (%)	98 (39)	101 (44)	.23
Invasive ventilation days, median (IQR)^a^	3.0 (4)	2.0 (3)	.60
Surgical patient, n (%)	118 (46)	88 (38)	.19
Diagnosis category			
Respiratory, n (%)	88 (35)	72 (31)	.44
Elective postsurgical, n (%)	89 (35)	72 (31)	.38
Cardiac, n (%)	17 (7)	30 (13)	.02
Neurological, n (%)	13 (5)	16 (7)	.40
Trauma, n (%)	29 (11)	12 (5)	.01
Sepsis, n (%)	2 (1)	6 (3)	.12
Metabolic, n (%)	4 (2)	7 (3)	.28
Other, n (%)	12 (5)	15 (7)	.29
Admission			
ICU length of stay in days, median (IQR)	2.0 (3)	2.0 (2)	.82
24 h to 7 d, n (%)	224 (88)	209 (91)	.34
Medication during ICU admission			
Prescriptions, median (IQR)	12.5 (20)	15.0 (19)	.46
>5 prescriptions, n (%)	203 (80)	202 (88)	.02
Administrations, median (IQR)	21.0 (40)	22.0 (38)	.81
Patient with high‐risk medication, n (%)	171 (67)	161 (70)	0.52

Abbreviations: ICU, intensive care unit; IQR, interquartile range; PRISM III, Pediatric Risk of Mortality Score III.

a Calculated for patient with invasive ventilation.

### 3.2 Medication errors

**Figure 2:**
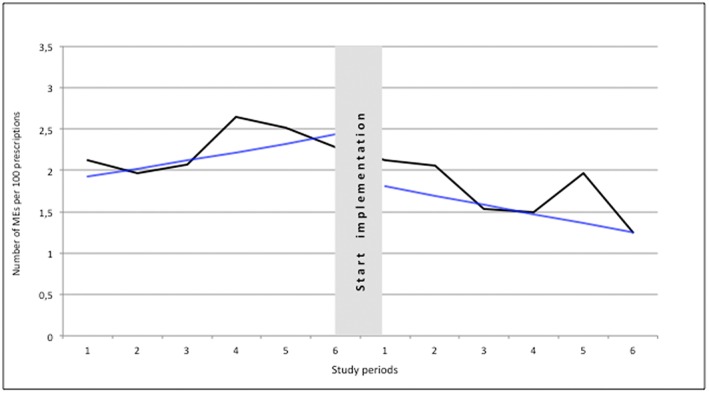
Medication errors (MEs) per 100 prescriptions during the study periods

We identified 153 MEs in the preintervention period, corresponding to 2.27 per 100 prescriptions, and 90 MEs in the postintervention period, corresponding to 1.74 per 100 prescriptions. Autoregressive integrated moving average analyses showed a stable incidence of MEs during the preintervention period (β = .10; 95% CI, −0.03 to 0.23; *P* = .11). We observed a significant change in the slopes between the preintervention and postintervention periods (β = −.21; 95% CI, −0.41 to ‐0.02; *P* = .04). Immediately after the start of the intervention, we observed a statistically nonsignificant decrease of 0.61 MEs per 100 prescriptions (β = −.61; 95% CI, −1.31 to 0.08; *P* = .07), corresponding to 23% reduction of MEs. These results are corrected for the significant difference between the preintervention group and postintervention group at baseline: patients with more than 5 prescriptions. The results are visually presented in Figure 2. Parameter estimates are summarized in Table 2.

**Table 2:** Interrupted time series analysis

MEs Per 100 Prescriptions	β (SE)	95% CI	*P* Value
Intercept (β0)	1.92		
Slope preintervention (β1)	.10 (0.05)	−0.03 to 0.23	.11
Slope postintervention	−.11 (0.06)	−0.25 to 0.02	.08
Slope differences (β3)	−.21 (0.07)	−0.41 to −0.02	.04
Level change directly after intervention (β2)	−.61 (0.28)	−1.31 to 0.08	.07
Relative effect directly after intervention	23%		

Abbreviation: ME, medication error. β1 estimates the preintervention slope. β2 estimates the difference between the observed level just after the intervention started and that predicted by the preintervention slope. β3 estimates the difference in slopes between the preintervention and postintervention periods.

We categorized the identified MEs in different types of error, eg, omission, dosage, or monitoring error. In addition, the stage of medication process in which the MEs occurred was identified. In the preintervention period, 133/153 MEs (87%) were categorized as prescribing errors (87%), as opposed to 82/90 (87%) in the postintervention period. Omissions of prescriptions and errors in dosages were common types of error. An overview of the results is presented in Table 3.

**Table 3:** Characteristics of the medication errors

Characteristic	Preintervention, 153 MEs	Postintervention, 90 MEs	*P* Value
Medication process			
Prescription, n (%)	133 (87)	82 (91)	.32
Administering, n (%)	11 (7)	3 (3)	.21
Monitoring, n (%)	8 (5)	5 (6)	.91
Preparation, n (%)	1 (<1)	0	.44
Type of ME			
Omission, n (%)	91 (60)	43 (48)	.08
Dosage, n (%)	25 (16)	31 (34)	.01
Monitoring error, serum concentration, n (%)	7 (5)	5 (6)	.73
Other, n (%)	30 (19)	11 (12)	.76
High‐risk medication			
High‐risk medication involved in MEs, n (%)	21 (14)	13 (14)	.23
Consequences for patients			
No consequences, n (%)	130 (85)	84 (93)	.08
Temporary harm, requiring intervention, n (%)	17 (11)	6 (7)	.25
Temporary harm, prolonged PICU stay, n (%)	6 (4)	0	.14
Permanent harm, life threatening or fatal	0	0	1.00

Abbreviations: ME, medication error; PICU, pediatric intensive care units.

### 3.3 Patient harm

Of the 153 MEs that had occurred in the preintervention period, we identified 23 harmful MEs (15%), corresponding to 0.34 per 100 prescriptions. In the postintervention period, 6 out of 90 MEs (7%) were identified as harmful, corresponding to 0.11 per 100 prescriptions. Autoregressive integrated moving average analyses revealed no statistically significant differences in the slopes between the preintervention and postintervention periods (β = −.01; 95% CI, −0.17 to 0.17; *P* = .88). Also, no statistically significant differences were found in the number of harmful MEs in the postintervention period directly following the intervention (β = −.07; 95% CI, −0.67 to 0.53; *P* = .79).

The experts classified the observed harm as temporary and requiring intervention in 23 harmful MEs (79%) and temporary with prolonged PICU hospitalization in 6 harmful MEs (21%).

### 3.4 Recommendations made by the clinical pharmacist

During the postintervention period, 230 intensive care patients were admitted to the PICU and 75 patients were audited (33%). The clinical pharmacists made 147 recommendations. The most common types of recommendations were dose adjustment (32%), discontinuation of a medication (23%), and monitoring of serum concentrations (22%). Of the 147 recommendations, 63% were accepted and given a follow‐up within 24 hours. Another 28% of the recommendations were seriously considered but not accepted for various reasons (eg, the patient's situation had changed). No follow‐up was given to 9% of the recommendations without reason. Examples of recommendations are presented in Table 4.

**Table 4:** Recommendations by the clinical pharmacist

Recommendations	n = 147	Examples
Dose adjustment, n (%)	47 (32)	Decrease dose of omeprazol, according to age < 1 y
		Increase dose of paracetamol, according to weight > 40 kg
Drug discontinuation, n (%)	34 (23)	Stop potassium in case of hyperkalemia
		Stop antibiotics after bacteriology culture came back negative
Monitoring, serum concentration, n (%)	32 (22)	Monitor gentamicin serum levels
		Monitor lactate levels in case of high‐dosage propofol
Start new drug, n (%)	18 (12)	Start antiepileptic drug after unintentional discontinuation (home medication)
		Start vitamins D and K in newborn
Administration, n (%)	7 (5)	Switch of total parenteral nutrition to central venous catheter
Others, n (%)	9 (6)	Correct prescription after confusion between prednisolone and methylprednisolone

## 4 DISCUSSION

Our study shows that the implementation of structured medication audit, followed by timely feedback by a clinical pharmacist as part of the multidisciplinary team, resulted in a significant reduction of MEs in a tertiary PICU. We observed a nonsignificant decrease in medication‐related patient harm. The proactive role of the clinical pharmacist resulted in recommendations with a high acceptance rate.

We identified only 1 previous high‐quality study that investigated interventions by a clinical pharmacist on a PICU.^14^ This study of Kaushal et al reported a reduction of serious MEs on a PICU from 29 to 6 per 1000 patient days after the introduction of a clinical pharmacist. However, in that study, the definition of MEs differed from our broader definition. In addition, the clinical pharmacist was present full time on the PICU, while in our study the pharmacist spent approximately 3 h/day on the PICU. Our study demonstrates that a comparable decrease in the incidence of MEs after the introduction of a clinical pharmacist can be achieved also with a more cost‐effective protocol. Other studies that have investigated the effect of the presence of a clinical pharmacist on a PICU involved single‐arm designs without a control group and focused on the recommendations and their acceptance by doctors and nurses rather than on the reduction of MEs.^19^, ^20^, ^21^, ^22^, ^23^ Our finding that most recommendations of the clinical pharmacist concerned dosages is in accordance with the aforementioned studies, but the acceptance rates of the recommendations of 95% and 98% were higher than the 63% acceptance rate in our study.^19^, ^20^, ^23^

In our study, the clinical pharmacist was actively involved in the medication process of 1 to 2 patients per day, who were considered most at risk for MEs. We performed a post hoc analysis to explore differences in the prevalence of MEs between patients whose medications were audited and discussed in the PICU team and patients without the medication audit. This analysis showed a significant difference between the 2 groups (mean difference = −1.71; 95% CI, −3.13 to −0.28; *P* = .03), meaning the prevalence of MEs per 100 prescriptions is significantly lower in patients with medication audit than those without. This result suggests that the intervention has no effect (or a delayed effect) in the nonaudited patients, but this hypothesis must be investigated in future research.

We found no significant effect of the interventions of the clinical pharmacist on patient harm. This might be explained by the low baseline rate of harmful MEs, and our study may have been underpowered to detect a difference. Although the low number of harm incidents is consistent with previous studies (6.9), these results may be underestimated as we studied patient harm during the stay on the PICU only, and we did not perform a follow‐up after transfer or discharge.

It can be expected that in the future computerized physician order entry systems will increasingly support the medication prescription process, possibly marginalizing the role of the clinical pharmacist. Although a computerized physician order entry reduces MEs in children,^24^, ^25^ it is important to note that information technology introduces new errors.^26^ Ongoing research is necessary to determine if participation of a clinical pharmacist within the setting of a multidisciplinary team remains effective when the context changes. Also, future research might focus on the role of pharmacists in chronic disease management and medication therapy management. Economic evaluations suggest a cost avoidance effect of interventions by a clinical pharmacist, but robust comparative economic analyses are lacking.^27^, ^28^ Therefore, future research should focus on the economic costs and benefits of the participation of a clinical pharmacist on PICUs. Another direction for future research should focus on the risk factors that lead to MEs and related harm in critically ill children. Several risk factors have been studied, such as age, severity of illness, and surgery, but the existing studies are limited and report nonconclusive results.^8^, ^9^, ^29^, ^30^ Only the number of prescriptions seems to be an independent risk factor for MEs.^9^, ^31^

Our study was designed as a single‐center study. In such a setting and anticipating that the study intervention would influence behavior of the professionals and the organization of care, an ITS design is the recommended approach.^32^ The optimal number of data collection points is still under debate, with recommendations that vary from 3 to 12 points.^33^, ^34^, ^35^ We collected data at 6 points before and 6 points after the intervention, which is in line with the Cochrane Collaboration guidelines.^35^ An ITS does not provide protection against the effect of other events occurring at the same time as the study intervention. A comparable patient group that could be used as a control group was not available at our hospital. To increase the confidence in the study results, we studied the rate of safety incidents during the study periods as a control variable. This analysis shows no significant differences between the preintervention and postintervention periods (β2 = .16; 95% CI, −0.03 to 0.36; *P* = .11 and β3 = .03; 95% CI, −0.02 to 0.08; *P* = .18). Also, the capacity of both nursing and medical staffing, a known risk factor for adverse events, was stable.^36^, ^37^

We recognize several limitations in our study. First, because of limited resources, the clinical pharmacist was present from Monday to Friday. We are aware that patients on a PICU may be instable and that relevant changes in medications are to be expected also during the weekends. The inclusion of patients that were admitted to the PICU during the weekends might have resulted in an underestimation of our results. Second, we retrospectively reviewed clinical records to detect potential MEs (harmful or otherwise). The results of this method depended on the information documented by doctors and nurses, which might have introduced an underestimation of MEs.^38^ Third, blinding of the researchers was not complete during the process of identification of MEs, since the researchers knew whether the patient had been admitted during the preintervention or postintervention period. However, both researchers and experts were completely blinded for the presence of an audit by the clinical pharmacist. Finally, this research was performed in a single‐center study. Although generalizability of the results might be limited, our study clearly shows an increase in drug safety in our setting after the introduction of a medication audit by a clinical pharmacist. The authors are aware that some excellent institutions already have 24/7 coverage by a clinical pharmacist. However, depending on existing local prescription procedures, patient population, resources, and pharmacological staffing, our results may be of interest for other health care settings around the globe that are similar to our situation.

## CONFLICT OF INTERESTS

The authors declare no conflict of interest.

## AUTHOR CONTRIBUTIONS

Conceptualization: Jolanda Maaskant, Marieke Tio, Reinier van Hest, Hester Vermeulen, Vincent Geukers

Data curation: Jolanda Maaskant, Marieke Tio

Formal Analysis: Jolanda Maaskant, Marieke Tio

Investigation: Jolanda Maaskant, Marieke Tio

Methodology: Jolanda Maaskant, Marieke Tio, Reinier van Hest, Hester Vermeulen, Vincent Geukers

Supervision: Hester Vermeulen, Vincent Geukers

Project administration: Jolanda Maaskant, Marieke Tio

Writing ‐ reviewing and editing: Jolanda Maaskant, Marieke Tio, Hester Vermeulen

Writing ‐ original draft: Jolanda Maaskant, Marieke Tio, Reinier van Hest, Hester Vermeulen, Vincent Geukers

## Supporting information


**Appendix S1**
Definitions and classifications in severity of medication errors (NCC MERP)

**Appendix S2**
Medication audit form
