# Supplementary material for: Medication audit and feedback by a clinical pharmacist decrease medication errors at the PICU: An interrupted time series analysis
Source: Health Sci Rep. 2018 Jan 19;1(3):e23. doi: 10.1002/hsr2.23 (PMC6200092; doi:10.1002/hsr2.23)
Supplement: Supplementary file 1 — Appendix S1 Definitions and classifications in severity of medication errors (NCC MERP) [file HSR2-1-e23-s001.docx]

**Appendix 1 Definitions and classifications in severity of medication errors (NCC MERP)**

| Medication error |
| --- |
| Any preventable event that may cause or lead to inappropriate medication use or patient harm while the medication is in the control of the health care professional, patient or consumer. Such events may be related to professional practice, health-care products, procedures and systems, including prescribing; order communication; product labeling; packaging and nomenclature; compounding; dispensing; distribution; administration; education; monitoring and use. |
| Harmful medication error |
| Any medication error with potential for patient harm, but no patient harm occurred for whatever reason, e.g. the error was intercepted before it reached the patient or the error reached the patient but did not result in patient harm. |
| Harm |
| Temporary or permanent impairment of the physical, emotional, or psychological function or structure of the body and/or pain resulting from this impairment, which requires intervention. |

| No error |  |
| --- | --- |
| A: | Circumstances or events that have the potential to cause error. |
| Error, no harm |  |
| B: | An error occurred, but the error did not reach the patient (an “error of omission” does reach the patient). |
| C: | An error occurred that reached the patient but did not cause patient harm. |
| D: | An error occurred that reached the patient and required monitoring to confirm that it resulted in no harm to the patient and/or required intervention to preclude harm. |
| Error, harm |  |
| E: | An error occurred that may have contributed to or resulted in temporary harm to the patient and required intervention. |
| F: | An error occurred that may have contributed to or resulted in temporary harm to the patient and required initial and prolonged hospitalization. |
| G: | An error occurred that may have contributed to or resulted in permanent patient harm. |
| H: | An error occurred that required intervention to sustain life. |
| I: | An error occurred that may have contributed to or resulted in patient death. |
