# Supplementary material for: Medication audit and feedback by a clinical pharmacist decrease medication errors at the PICU: An interrupted time series analysis
Source: Health Sci Rep. 2018 Jan 19;1(3):e23. doi: 10.1002/hsr2.23 (PMC6200092; doi:10.1002/hsr2.23)
Supplement: Supplementary file 2 — Appendix S2 Medication audit form [file HSR2-1-e23-s002.docx]

**Appendix 2 Medication audit form**

Date audit: ………………………………………………………………………..

Name patient: ………………………………………………………………………..

Patient hospital number: ………………………………………………………………………..

Date of birth: ………………………………………………………………………..

Weight in kg.: ………………………………………………………………………..

Reason admission: ………………………………………………………………………..

Known allergies, please specify: ………………………………………………………………………..

Dialysis (CVVH, HD, CAPD): ………………………………………………………………………..

Actual problems: ………………………………………………………………………..

Othere relevant information: ………………………………………………………………………..

| **Lab. value, clinical signs** | **Present value and trend**  **(↑ or ↓)** | **Reference values** | | |
| --- | --- | --- | --- | --- |
|  |  | **0 - 2 wks** | **0 - 1 yr** | **1 - 18 yrs** |
| Creatinine (micromol/l) |  | - | 35 - 80 | 35 - 100 |
| ASAT (U/l) |  | <125 | <95 | 1 – 3 yrs <60  3 – 15 yrs <40 |
| ALAT (U/l) |  | <65 | <65 | <45 |
| Albumin (g/l) |  | 29 - 55 | 1 - 4 months : 28 - 50  4 months - 1 yr: 39 - 51 | 37 - 55 |
| Bilirubin total (micromol/l) |  | 0 - 24 hrs: 34 - 103  24 - 48 hrs:  103-120  3 - 5 days: 68 - 205 | <17 | <17 |
| Hemoglobin (mmol/l) |  | 0 - 7 days: 8.5 - 12.5  8 - 14 days: 6 - 9 | 6 - 9 | 1 - 7 yrs: 6 - 9  7 - 16 yrs: 6.5 - 10 |
| Trombocytes (10E9/l) |  | 150 - 600 | 150 - 600 | 1 - 7 yrs: 150 - 600  7 - 16 yrs: 150 - 450 |
| Leukocytes (WBC) (10E9/l) |  | 0 - 7 days: 12 - 24  8 - 14 days: 6 - 17 | up to 0.5 yr: 6 - 17  0.5 – 1 yr: 4 - 16 | 1 - 3 yrs: 4 - 16  3 - 7 yrs: 4 - 15  7 - 16 yrs: 4 - 14 |
| pH |  | 0 - 7 days:  7.33 - 7.49  8 – 14 days:  7.32 – 7.43 | up to 2 months:  7.32 - 7.43  2 - 12 months:  7.34 - 7.46 | 7.35 - 7.45 |
| CRP (mg/l) |  | <5 | <5 | <5 |
| APTT (sec) |  | 22 - 30 | 22 - 30 | 22 - 30 |
| Glucose (mmol/l) |  | 0 - 1 days: 2.2 - 3.3  2 - 14 days:  2.8 - 4.5 | 2.8 - 4.5 | 1 – 15 yrs: 3.3 - 5.6  15 – 18 yrs: 4.1 - 5.6 |
| Sodium (mmol/l) |  | 133 - 142 | 0 - 0.5 yrs: 133 - 142  0.5 - 1 yrs: 135 - 145 | 135 - 145 |
| Potassium (mmol/l) |  | 4 - 6 | 0 - 0.5 yrs: 4 - 6  0.5 - 1 yrs: 3.7 - 5.6 | 3.5 - 5 |
| Temperature (°C) |  | 36.5 - 37.5 | 36.5 - 37.5 | 36.5 - 37.5 |
| Diuresis (ml/kg/h) |  | 2 | 2 | up to 25 kg: 2  >25 kg: 1 |
| Comfort score |  | 11 - 17 | 11 - 17 | 11 - 17 |
| Abstinence score (SOS) |  | <5 | <5 | <5 |
| Blood pressure (mmHg) |  | 55 | up to 0.5 yr: 60 - 65  0.5 – 1 yr: 65 | 1 – 6 yrs: 65 - 70  6 -12 yrs: 75  12 – 18 yrs: 80 |
| Heartrate (/min) |  | 90 - 180 | tot 0.5 yr: 100 – 180  0.5 – 1 yr: 93 - 168 | 1 – 3 yrs: 76 – 156  3 – 6 yrs: 65 – 136  6 -12 yrs: 52 – 123  12 – 18 yrs: 43 - 108 |
| Defaecation |  | 7x/day - 1x/wk | 7x/day - 1x/wk | 3x/day - 1x/3 days |
| Gastric or duodenum tube? | yes / no | - | - | - |

**Usage of lorazepam and/or methadon to prevent abstinence syndrome?**

Last daily dose midazolam (mg/kg/h): …………………… = …………….. mg/day.

Stopping date: ……………………………………………..

Last daily morphine/phentanyl (microg/kg/h): ………………. = …………….. mg/day.

Stopping date: ……………………………………………..

**Usage of antibiotics?**

Name antibiotic: ……………………………………………………. during (no of days): ……………….

Name antibiotic: ……………………………………………………. during (no of days): ……………….

Name antibiotic: ……………………………………………………. during (no of days): ……………….

Name antibiotic: ……………………………………………………. during (no of days): ……………….

**Results of culture/PCR?**

Date culture: …………………..micro organism: ……………………preferred treatment:……………….

Date culture: …………………..micro organism: ……………………preferred treatment:……………….

Date culture: …………………..micro organism: ……………………preferred treatment:……………….

Date culture: …………………..micro organism: ……………………preferred treatment:……………….

1 = <1 yr; 2 = 1-8 yrs; 3 = >8 yrs

^*^ = high risk medication

|  | **Present dose (mg/kg/day in x doses)** | **Dose and monitoring adequate?** | **Alert?** | **Adverse event?** | **Advice, remarks.** |
| --- | --- | --- | --- | --- | --- |
| Check doses inotropics:  Noradrenalin^*^  Adrenalin^*^  Dopamin^*^  Dobutamin  Salbutamol |  | yes / no  yes / no  yes / no  yes / no  yes / no | Hypertension:  1. ABPm>65  2. ABPm>75  3. ABPm>80  Tachycardia:  1. >180  2. >156  3. >123  Potassium:  1. <3.7  2+3. <3.5 | yes / no |  |
| Check doses Amiodaron^*^ |  | yes / no | Bradycardia:  1. <90  2. <65  3. <50  Hypotension:  1. ABPm<55  2. ABPm<65  3. ABPm<75  ALAT / ASAT:  1. >65 / >125  2. >45 / >60  3. >45 / >40  Bilirubin:  1+2+3. >17^2^  TSH:  1+2+3: <0.5, >5 | yes / no |  |
| Check doses digoxin^*^  Attention:  Renal clearance  Serum concentration  Interaction with Macrolides, Itraconazol or Ketoconazol |  | yes / no  Doses adapted to reduced renal clearance?  yes / no / n.a.  Serum concentration planned?  yes / no / n.a.  Advice accepted?  Yes / no / n.a.  Potassium normal: (3.5<K<5 mmol/l)  yes / no | Bradycardia:  1. <90  2. <65  3. <50 | yes / no |  |
| Check serum glucose  or  Check doses insuline^*^ |  | yes /no | Glucose:  1. <2.8, >6.1  2. <3.3, >6.1  3. <4.1, >6.1  Potassium:  1. <3.7  2+3. <3.5 | yes / no |  |
| Check doses sedatives:  Propofol^*^  Clonidine  Midazolam  Esketamine  Lorazepam  Chloralhydrate  ­­­____________  Attention:  Indication and schedule of tapering dosages. |  | yes / no  yes /no  yes / no  yes / no  yes / no  yes / no  yes / no | Bradycardia:  1. <90  2. <65  3. <50  Hypotension:  1. ABPm<55  2. ABPm<65  3. ABPm<75  Triglycerides:  1. >2.2  2+3. >5  LDL:  1+2: >3.63  3: >3.52  pH:  1: <7.32  2+3. <7.35  Comfort score:  1+2+3. <11/ >17  Abstinence score 1+2+3. >8 | yes / no |  |
| Check doses opiates^*^:  Fentanyl  Morfine  Bupivacain/ sufentanil  Methadon  ____________  Attention:  Indication and schedule of tapering dosages. |  | yes / no  yes / no  yes / no  yes / no  yes / no  Laxatives started?  yes /no | Hypotension:  1. ABPm<55  2. ABPm<65  3. ABPm<75  Bradycardia:  1. <90  2. <65  3. <50  Comfort score:  1+2+3. <11/ >17^$3^  Abstinence score 1+2+3. >5  Defaecation:  1: <1x/week  2+3: <1x/3 days | yes / no |  |
| Check doses antithrombotics:  Heparin^*^  Nadroparin  Alteplase  Attention:  Renal clearance |  | yes / no  yes / no  yes / no  Doses adapted to reduced renal clearance?  yes / no / n.a. | APTT:  1+2+3. >30  Trombocytes:  1+2+3. <150  ALAT / ASAT:  1. >65 / >125  2. >45 / >60  3. >45 / >40 | yes / no |  |
| Check doses antibiotics.  Attention:  Renal clearance  Serum concentration (Gentamicin^*^, Tobramycin, Amikacin, Vancomcyin). |  | Yes /no  Doses adapted to reduced renal clearance?  yes / no / n.a.  Serum concentration planned:  yes / no / n.a.  Advice accepted?  yes / no / n.a.  Indication for culture-based change of antibiotic?  yes / no / n.a.  Change to oral antibiotic possible?  yes / no / n.a.  Signs of infection present (fever, WBC, CRP, culture)?  yes / no | Creatinine:  1+2+3. >100  Diuresis:  1+2+3. <0.5  WBC:  1. <4, >17  2. <4, >15  3. <4, >14  Hb:  1+2. <6  3. <6.5  Trombocytes: 1+2+3. <150  ALAT / ASAT:  1. >65 / >125  2. >45 / >60  3. >45 / >40  Comfort score:  1+2+3. <11 | yes /no |  |
| Check doses antiviral medications:  (Val)aciclovir  (Val)ganciclovir  ____________  Attention:  Renal clearance |  | yes / no  Doses adapted to reduced renal clearance?  yes / no / n.a. | Creatinine:  1+2+3. >100  Diuresis:  1+2+3. <0.5  WBC:  1. <4, >17  2. <4, >15  3. <4, >14  Hb:  1+2. <6  3. <6.5  Trombocytes: 1+2+3. <150  ALAT / ASAT:  1. >65 / >125  2. >45 / >60  3. >45 / >40  Convulsions | yes / no |  |
| Check doses antifungal medications:  Fluconazol  Flucytosine  ____________  Attention:  Renal clearance |  | yes / no  Doses adapted to reduced renal clearance?  yes / no / n.a.  Serum concentration planned:  yes / no / n.a.  Advice accepted?  yes / no / n.a.  Signs of infection still present (fever, WBC, CRP, culture)?  yes / no | ALAT / ASAT:  1. >65 / >125  2. >45 / >60  3. >45 / >40  Bilirubin:  1+2+3. >17  WBC:  1. <4, >17  2. <4, >15  3. <4, >14  Trombocytes: 1+2+3. <150  Convulsions | yes / no |  |
| Check doses immunosuppressiva^*^:  Cyclosporine  Tacrolimus^*^  Sirolimus  Mycophenolic acid  Azathioprine  Attention interaction azathioprine - allopurinol |  | yes / no  Serum concentration planned:  yes / no / n.a.  Advice accepted?  yes / no / n.a. | Creatinine:  1+2+3. >100  Diuretics:  1+2+3. <0.5  WBC:  1. <4^1^, >17  2. <4, >15  3. <4, >14  Hb:  1+2. <6  3. <6.5  Trombocytes: 1+2+3. <150  Hypertension:  1. ABPM>65  2. ABPM>75  3. ABPM>80  Glucose:  1. >4.5  2+3. >5.6  LDL:  1+2: >3.63  3: >3.52 | yes / no |  |
| Check doses orale cytostatica^*^:  Methotrexate  Etoposide  Melfalan  ____________  Attention:  Renal clearance |  | yes /no  Doses adapted to reduced renal clearance?  yes / no / n.a.  Interaction?  yes / no | WBC:  1. <4, >17  2. <4, >15  3. <4, >14  Hb:  1+2. <6  3. <6.5  Trombocytes: 1+2+3. <150  ALAT / ASAT:  1. >65 / >125  2. >45 / >60  3. >45 / >40  Bilirubin:  1+2+3. >17^2^ | yes / no |  |
| Check doses antiepileptics:  Phenytoin^*^  Valproic acid  Carbamazepin  Levetiracetam  Phenobarbital  ____________ |  | yes / no  Serum concentration planned:  yes / no / n.a.  Advice accepted?  yes / no / n.a.  Interaction?  yes / no | Comfort score:  1+2+3. <11  Tachycardia:  1. >180  2. >156  3. >123  Hypotension:  1. ABPm<55  2. ABPm<65  3. ABPm<75  WBC:  1+2+3. <4  Hb:  1+2. <6  3. <6.5  Trombocytes: 1+2+3. <150  ALAT / ASAT:  1. >65 / >125  2. >45 / >60  3. >45 / >40  Bilirubin:  1+2+3. >17  Ammonia (NH_3_):  1. <2wkn >90  1+2+3. >50 | yes / no |  |
| Total Parenteral Nutrition (TPN) | yes / no | Indication adequate?  yes / no | Triglycerides:  1. >2.2  2+3. >5  LDL:  1+2: >3.63  3: >3.52 | yes /no |  |
| Start potassium supplement or RAS-inhibitor and congestive failure / reduced renal function / arrhythmias / diabetes / combination with Spironolacton / combination with diuretic / combination with Insulin / combination with Salbutamol | Check trend in potassium (last 3 values):  I. mmol/l  II. mmol/l III. mmol/l |  | Potassium  1. <½ yr:<4, >6  1. >½ yr:<3.7,>5.6  2+3. <3.5, >5  Heart rate:  1. <90, >180  2. <65, >156  3. <50, >123  Arrythmia | yes / no |  |
|  |  | | **Alert?** | **Adverse event?** | **Advice, remarks.** |
| **SPECIFIC INTERACTIONS** | | | | | |
| QTc prolongations |  | | QTc:  1+2+3. >450 ms | yes / no |  |
|  |  | | **Adverse event?** | | **Advice, remarks.** |
| **ORGAN FUNCTION** | | | | | |
| Renal clearance  inadequate?  Creatinine:  1+2+3. >100^1^  Diuresis (oligury):  1. < 1 ml/kg/h  2+3. < 0.5 ml/kg/h | Check l all renally cleared medication. | | yes / no | |  |
| CVVH of HD | Check all medication that might interact with renal replacement therapy. | | yes /no | |  |
| Hepatic clearance inadequate?  ALAT / ASAT:  1. >65 / >125  2. >45 / >60  3. >45 / >40  Bilirubin:  1+2+3. >17 | Check all hepatically cleared medication. | | yes / no | |  |
| Defaecation?  1: <1x/week  2+3: <1x/3 days | Attention: opiates, calciumantagonisten, tricyclische antidepressiva, anticholinergica. | | yes / no | | Advice to start laxatives. |
| Gastro-enteritis, bowel disturbances? | Attention: absorption of oral medications. | | yes / no | |  |
| **OTHERS** | | | | | |
| General | Check dosages of all medications | | yes / no | |  |
| Medication omission?  Attention: home medication. | yes / no  please specify: | | yes / no | |  |
| Medication through gastric or duodenum tube? | yes / no  Check if medication are suitable for administration through a tube. | | yes / no | |  |
| Nutrition through duodenum tube? | Proton-pump inhibitor started? | | yes / no | |  |
| Intravenous medication? | yes / no  Check:  Pump rates  Labelling  Administration through periferal or central venous catheter conform protocol | | yes / no | |  |

**Summary and advices to be discussed with the PICU team.**

| **Advices** | **Advice accepted?** | **Reason for non- acceptance?** | **Follow-up and evaluation** | **Advice executed within 24 hrs?** |
| --- | --- | --- | --- | --- |
|  | yes / no |  |  | yes / no |
|  | yes / no |  |  | yes / no |
|  | yes / no |  |  | yes / no |
|  | yes / no |  |  | yes / no |
|  | yes / no |  |  | yes / no |
|  | yes / no |  |  | yes / no |
|  | yes / no |  |  | yes / no |
